# FAD012, a Ferulic Acid Derivative, Preserves Cerebral Blood Flow and Blood–Brain Barrier Integrity in the Rat Photothrombotic Stroke Model

**DOI:** 10.3390/biomedicines13102403

**Published:** 2025-09-30

**Authors:** Hiroshi Sugoh, Hirokazu Matsuzaki, Jun Takayama, Naohiro Iwata, Meiyan Xuan, Bo Yuan, Takeshi Sakamoto, Mari Okazaki

**Affiliations:** 1 Laboratory of Pharmacology, Graduate School of Pharmaceutical Sciences, Josai University, Saitama 350-0295, Japan; gyd2202@josai.ac.jp (H.S.); ma-tsu@josai.ac.jp (H.M.); yuanbo@josai.ac.jp (B.Y.); 2 Laboratory of Organic and Medicinal Chemistry, Graduate School of Pharmaceutical Sciences, Josai University, Saitama 350-0295, Japan; takayama@josai.ac.jp (J.T.); genbien@josai.ac.jp (M.X.); sakamoto@josai.ac.jp (T.S.); 3 Laboratory of Immunobiochemistry, Graduate School of Pharmaceutical Sciences, Josai University; Saitama 350-0295, Japan; n-iwata@josai.ac.jp

**Keywords:** 3,5-dimethyl-4-hydroxycinnamic acid (ferulic acid derivative 012; FAD012), ferulic acid (FA), photothrombotic stroke, brain infarction, tight junction (TJ) protein, cerebral vascular endothelial cells, blood–brain barrier (BBB), cerebral blood flow (CBF), endothelial nitric oxide synthase (eNOS)

## Abstract

**Background/Objectives:** The rapid progression of stroke often results in irreversible brain damage and poor outcomes when treatment is delayed. Prophylactic administration of FAD012 (3,5-dimethyl-4-hydroxycinnamic acid), a synthetic derivative of ferulic acid (FA), has demonstrated cerebroprotective effects in ischemic models through antioxidant and endothelial protective mechanisms. This study investigated the effects of FAD012 on cerebral infarction and blood–brain barrier (BBB) integrity using a photothrombotic stroke model in rats. **Methods:** Male Sprague Dawley rats received a single intraperitoneal injection of FAD012 or FA (100 or 300 mg/kg) 60 min prior to stroke induction. Under isoflurane anesthesia, the middle cerebral artery was exposed, and stroke was induced by intravenous administration of Rose Bengal followed by green laser irradiation. Cerebral blood flow (CBF) was monitored by laser Doppler flowmetry. BBB disruption was evaluated by Evans Blue extravasation and immunohistochemistry for tight junction (TJ) proteins. **Results:** Control rats exhibited extensive infarction, BBB disruption, and reduced expression of claudin-5, occludin, and ZO-1, along with fragmented collagen IV. In contrast, FAD012 (300 mg/kg) significantly attenuated CBF reduction, reduced infarct size, preserved BBB integrity, and maintained TJ protein expression, with greater efficacy than an equivalent dose of FA. FAD012 also preserved the expression and phosphorylation of endothelial nitric oxide synthase (eNOS), a key marker of vascular integrity. The CBF-preserving effect of FAD012 was completely abolished by N^G^-nitro-L-arginine methyl ester (L-NAME), a nitric oxide synthase inhibitor. **Conclusions:** These findings suggest that FAD012 protects endothelial function, thereby contributing to the maintenance of CBF and BBB integrity, supporting its potential as a prophylactic therapeutic agent for ischemic stroke.

## 1. Introduction

Ischemic stroke is a major global health concern, particularly among the elderly and closely associated with lifestyle-related conditions such as hypertension, diabetes, and hyperlipidemia [1,2,3]. The lifetime risk of stroke is estimated to be approximately 25%, and it remains the third leading cause of long-term disability worldwide due to its profound impact on cognitive, motor, and sensory functions [4]. With global population aging, the socioeconomic burden of stroke is expected to increase substantially in the coming decades [5].

Currently, thrombolytic therapy with tissue plasminogen activator (t-PA) remains the only pharmacological treatment for acute ischemic stroke approved by the U.S. Food and Drug Administration [6]. Although thrombolytic therapy with t-PA is effective in re-establishing cerebral blood flow (CBF) via enzymatic thrombus dissolution [7,8], the clinical applicability of t-PA is limited to only 5–8% of patients due to its narrow therapeutic time window (4.5 h) and stringent eligibility criteria. Moreover, t-PA carries a substantial risk of hemorrhagic complications and exhibits only modest therapeutic efficacy (~30%) [9,10,11,12]. Among the mechanisms underlying these limitations, disruption of the blood–brain barrier (BBB) is particularly critical, as it not only shortens the therapeutic window but also increases the risk of hemorrhagic transformation [9,10]. Consequently, novel therapeutic strategies aimed at preserving BBB integrity following ischemic stroke may substantially expand the safe and effective use of t-PA. Given the sudden and unpredictable onset of stroke, there is an urgent need to develop prophylactic agents that can be administered safely to high-risk individuals prior to stroke occurrence. Such agents, if available, would ideally preserve neurovascular integrity at the time of ischemic insult, thereby mitigating subsequent brain injury and contributing to improved clinical outcomes.

**Figure 1:**
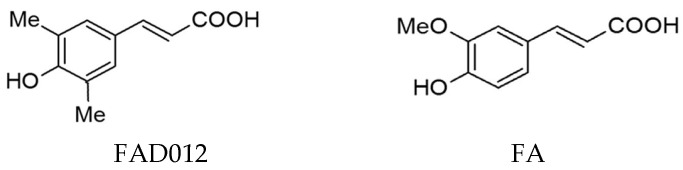
Chemical structures of 3,5-dimethyl-4-hydroxycinnamic acid (FAD012) and ferulic acid (FA, 4-hydroxy-3-methoxycinnamic acid).

To address this issue, we focused on ferulic acid (FA), a naturally occurring phenolic compound with well-documented antioxidant, anti-inflammatory, and antiplatelet properties [13,14]. Building upon these characteristics, we synthesized a series of FA derivatives and identified FAD012 (3,5-dimethyl-4-hydroxycinnamic acid; Figure 1) as one of the most promising candidates based on its superior neuroprotective efficacy. Compared to FA, FAD012 exhibits increased lipophilicity and enhanced electron-donating capacity, which confer greater antioxidant and cytoprotective activity with minimal toxicity. In a rat model of middle cerebral artery occlusion following reperfusion (MCAO/Re), FAD012 preserved endothelial nitric oxide synthase (eNOS) expression, thereby sustaining CBF via nitric oxide (NO)-mediated vasodilation. This was accompanied by significant reductions in neurological deficits and infarct size [15]. In vitro, FAD012 markedly attenuated hydrogen peroxide (H_2_O_2_)-induced cytotoxicity in rat brain microvascular endothelial cells (RBMVECs), further demonstrating its superior protection against oxidative stress-induced endothelial injury compared to FA [15]. Collectively, these findings suggest that FAD012 possesses both prophylactic and cerebroprotective potential in the context of ischemic stroke [15,16].

Anatomically, brain microvascular endothelial cells form tight junctions (TJs) that seal the intercellular space and establish the BBB. Along with pericytes, astrocytes, neuronal processes, perivascular microglia, and the basement membrane, these endothelial cells constitute the neurovascular unit [17,18]. As the gatekeeper of the central nervous system (CNS), the BBB plays a critical role in regulating paracellular permeability, ionic and metabolic homeostasis, nutrient transport, and cerebral hemodynamics [19]. Following ischemic stroke, neuroinflammation and oxidative stress induced by reactive oxygen species (ROS) consistently compromise BBB integrity, resulting in paracellular leakage of blood-derived components, vasogenic edema, hemorrhagic transformation, and increased mortality [20,21].

Given the potent endothelial protective properties of FAD012, we hypothesized that it may contribute to the preservation of BBB integrity under ischemic conditions. To test this hypothesis, we employed the photothrombotic stroke model, a well-established and reproducible model of focal ischemia. Unlike embolic or mechanical occlusion models, photothrombosis induces localized endothelial injury and platelet-rich thrombus formation via photoactivated ROS generation. In this model, illumination of intravenously administered Rose Bengal produces singlet oxygen, which oxidizes endothelial cell membranes and promotes platelet adhesion and aggregation, ultimately resulting in stable thrombus formation [22,23]. Moreover, because this model produces a permanent vascular occlusion without reperfusion, it closely mimics clinical situations in which recanalization therapy is not feasible. These pathophysiological characteristics make it particularly suitable for investigating acute-phase BBB disruption and cerebrovascular dysfunction in the acute phase of ischemia. In this study, we demonstrated that pretreatment with FAD012 delayed thrombus formation in the photothrombotic stroke rat model and attenuated ischemic brain injury by preserving CBF and protecting BBB integrity through the maintenance of endothelial function.

## 2. Materials and Methods

### 2.1. Animals

Male Sprague Dawley rats (7 weeks old, 210–230 g, *n* = 110) were obtained from Sankyo Labo Service Corporation (Tokyo, Japan). The animals were housed in a temperature-controlled environment (22 ± 1 °C) maintained at 22 ± 1 °C under a 12-hour light/dark cycle, with free access to standard chow (CE-2, CLEA Japan, Inc., Tokyo, Japan) and water. Following a one-week acclimation, the animals were randomly allocated to the experimental groups.

### 2.2. Treatment Schedule

FAD012 and FA were administered via intraperitoneal (i.p.) injection 60 min prior to stroke induction. Due to their poor aqueous solubility (predicted at approximately 0.5 mg/mL for FAD012 and 0.3 mg/mL for FA), both compounds were suspended in 0.5% carboxymethyl cellulose (CMC; Fujifilm Wako Chemical Co., Osaka, Japan) and administered intraperitoneally at a volume of 5 mL/kg body weight. Rats were randomly assigned to one of six experimental groups: a sham group (*n* = 16), which received 0.5% CMC without photothrombotic stroke induction; a CMC group (vehicle control, *n* = 23), administered 0.5% CMC followed by stroke induction; two FA (trans-4-hydroxy-3-methoxycinnamic acid, Sigma-Aldrich, St. Louis, MO, USA)-treated groups at doses of 100 mg/kg (*n* = 5) or 300 mg/kg (*n* = 19) followed by stroke induction; and two FAD012-treated groups at doses of 100 mg/kg (*n* = 5) or 300 mg/kg (*n* = 18) followed by stroke induction. The doses of FA and FAD012 (100 and 300 mg/kg, i.p.) were selected based on our preliminary experiments (unpublished data) and previous studies demonstrating neuroprotective effects of FA at 100–200 mg/kg via intravenous (i.v.) administration in ischemic models [24,25]. Considering that i.p. administration generally requires higher doses than i.v. injection, the use of 100 and 300 mg/kg was regarded as appropriate for evaluating the efficacy of FA and FAD012 under the present experimental conditions.

FAD012 was fully characterized by ^1^H and ^13^C NMR and mass spectrometry. The purity of FAD012, as determined by HPLC analysis, exceeded 95% [15,16].

To examine the involvement of NO in the action of FAD012 on CBF maintenance during ischemia, four additional groups were prepared: Sham + L-NAME (N^G^-nitro-L-arginine methyl ester; Sigma-Aldrich, St. Louis, MO, USA) group (*n* = 5), which received 0.5% CMC and L-NAME without photothrombotic stroke induction; and three groups subjected to photothrombotic stroke: CMC + L-NAME group (*n* = 5), FAD012 (300 mg/kg) + L-NAME group (*n* = 4), and FAD012 (300 mg/kg) + saline group (*n* = 5). L-NAME was administered intraperitoneally at a dose of 3 mg/kg, 30 min prior to stroke induction [26].

### 2.3. Photothrombotic Stroke Model

The photothrombotic stroke model was established as previously described [27,28,29]. Rats were anesthetized with isoflurane (5.0% for induction and 1.8–2.5% for maintenance; Viatris, Tokyo, Japan). A small incision was made in the left thigh to insert a catheter into the left femoral vein, which was then secured and sutured. To expose the middle cerebral artery (MCA), an incision was made between the left eye and ear, and the temporalis muscle along with surrounding tissue was carefully removed using an electrocautery device (Geiger thermal cautery unit, model 150, Delanco, NJ, USA). The mandibular process was excised, and a cranial window was created in the temporal bone using a dental drill (JSDA^®^ JD8500B, As One Corporation, Osaka, Japan).

**Figure 2:**
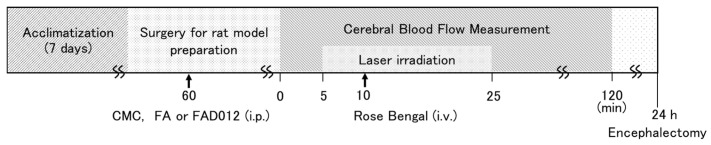
Experimental timeline for the photothrombotic stroke model. Abbreviations: CMC, carboxymethyl cellulose; i.v., intravenous; i.p., intraperitoneal.

Two additional cranial windows were drilled in the parietal regions of both hemispheres, through which cortical surface CBF was monitored for 120 min after stroke induction using a two-dimensional laser blood flow imaging system (OZ-2, LSI software ver. U40; LIA software ver. 4.0; Omega Wave Inc., Tokyo, Japan) [15,16]. Five mins after the start of CBF monitoring, a krypton laser (150 W light source, 20% output; L4887-13; Hamamatsu Photonics K.K., Hamamatsu, Japan) was applied to the target region for 20 min. After 5 min of laser irradiation, Rose Bengal (30 mg/kg; Fujifilm Wako Chemical Co., Osaka, Japan) was administered intravenously via the femoral catheter over 90 s period. Successful induction of thrombus formation and subsequent cerebral ischemia was confirmed by a sustained reduction in CBF for up to 120 min following laser irradiation (Figure 2). To evaluate the dynamics of thrombus formation, we measured the latency to maximal reduction in CBF following photothrombotic induction. The data were averaged at 5 min intervals, and the earliest time point at which CBF reached its minimum value was defined as the “elapsed time to CBF nadir”.

Following the completion of the 120 min monitoring session, the rats were anesthetized with urethane (1 g/kg, i.p.) (ethyl carbamate; Fujifilm Wako Chemical Co., Osaka, Japan) and maintained under deep anesthesia for an additional 24 h to minimize distress due to the invasive nature of the surgical procedure. The persistence of the thrombus for up to 24 h post-irradiation was confirmed by stereomicroscopic examination of brain tissue collected after euthanasia.

### 2.4. 2,3,5-Triphenyl Tetrazolium Chloride Staining

Cerebral infarct volume was assessed by 2,3,5-triphenyl tetrazolium chloride (TTC) staining as previously described [15]. Twenty-four hours after thrombus induction, the rats were euthanized, and brains were harvested and sliced into 2 mm thick coronal sections. The sections were incubated in phosphate-buffered saline (PBS) containing 2% TTC (Fujifilm Wako Chemical Co., Osaka, Japan) at 37 °C for 6 min. Infarct areas were identified by imaging analysis software (Image J, ver. 1.54d; National Institutes of Health, Bethesda, MD, USA). The infarct volume of each coronal slice (V) was estimated using the formula V = 2/3 × (rostral area + caudal area + √[rostral area × caudal area]). The values obtained from all slices were then summed to obtain the total infarct volume. The overall infarct volume (%) was calculated as: {[left hemisphere volume − (right hemisphere volume − infarct volume)] − left hemisphere volume} × 100.

### 2.5. Evans Blue Staining

To assess BBB integrity, Evans Blue (EB) staining was performed according to previously described methods with minor modifications [30,31]. EB (50 mg/kg, Fujifilm Wako Chemical Co., Osaka, Japan) was administered intravenously 23 h after stroke induction. One hour later, rats were euthanized, and brains were harvested, cut into 2 mm thick coronal sections, and photographed. The extent of EB extravasation was quantified using ImageJ software (ver. 1.54d) [32].

### 2.6. Immunohistochemistry

Immunofluorescence staining was performed as previously described [15] to evaluate the expression of endothelial and BBB-associated proteins, including TJ proteins (occludin, claudin-5, and ZO-1), collagen IV (a major component of the vascular basement membrane), and eNOS and its phosphorylated eNOS (p-eNOS; phosphorylated form at Ser1177). von Willebrand factor (vWF) was used as an endothelial marker to visualize cerebral microvessels [33]. Although ischemic injury may alter its distribution, in this study vWF labeling was mainly confined to vascular structures and was suitable for co-localization with tight junction proteins and eNOS. At 24 h after stroke induction, rats were euthanized, and brain tissues were collected and embedded in optimal cutting temperature compound to prepare frozen blocks. The tissues were rapidly frozen in an organic solvent mixture (pentane–hexane = 1:2) at −100 °C using a snap-freezing system (UT2000F; Leica, Bensheim, Germany). Coronal cryosections (10 μm thick) were prepared using a cryostat (CM3050S; Leica, Bensheim, Germany) and mounted onto glass slides. Sections were fixed in methanol for 1 min and subsequently washed with 0.01 M PBS. After blocking with Block Ace (DS Pharma Biomedical, Osaka, Japan) for 2 h at room temperature, sections were incubated overnight at 4 °C with the following primary antibodies: claudin-5 (1:200; mouse monoclonal, clone A-12, Santa Cruz Biotechnology, Dallas, CA, USA), occludin (1:200; mouse monoclonal, clone F-7, Santa Cruz Biotechnology, CA, USA), ZO-1 (1:200; rabbit polyclonal, clone 61-7300, Invitrogen, Waltham, MA, USA), collagen IV (1:500; rabbit polyclonal, clone 2150-1470, Bio-Rad, CA, USA), vWF (1:200; rabbit polyclonal, clone 27186-1-AP, Proteintech, IL, USA or 1:200; mouse monoclonal, clone C-12, Santa Cruz Biotechnology, CA, USA), eNOS (1:500; mouse monoclonal, clone A-9, Santa Cruz Biotechnology, CA, USA), and p-eNOS (Ser1177) (1:500; mouse monoclonal, clone 15E2, Santa Cruz Biotechnology, CA, USA). After three washes with PBS, sections were incubated for 2 h at room temperature with appropriate fluorescent secondary antibodies (e.g., Cy3- or FITC-conjugated IgG; 1:100; Chemicon International (Temecula, CA, USA) and Invitrogen (Waltham, MA, USA)). Nuclear counterstaining was performed with 4′,6-diamidino-2-phenylindole (DAPI; Cayman Chemical Company, Ann Arbor, MI, USA). Slides were mounted with 80% glycerol and coverslipped. Images were acquired using an upright fluorescence microscope (BX53; Olympus, Tokyo, Japan). Expression levels of each target protein in the cortex were quantified using MetaMorph software (ver. 7.8.10.0, Molecular Devices, San Jose, CA, USA). For TJ proteins, collagen IV, and eNOS, areas of fluorescence intensity exceeding a fixed threshold and colocalized with vWF, detected under constant laser settings, were defined as immunopositive and quantified accordingly. Histopathological evaluation was performed in a blinded manner, with the examiner unaware of the treatment groups. For each marker analyzed (claudin-5, occludin, ZO-1, and collagen IV), one representative coronal brain section was selected per animal for quantification. These markers were assessed in the same animal set. In contrast, eNOS and p-eNOS were evaluated in a separate cohort of animals. In all cases, statistical analyses were performed using biologically independent samples, where *n* refers to the number of animals per group, and each animal contributed a single data point per marker.

### 2.7. In Silico Absorption, Distribution, Metabolism, Excretion, and Toxicity Prediction

The absorption, distribution, metabolism, excretion, and toxicity (ADMET) profiles of FAD012 were predicted using two publicly available online tools: SwissADME (http://www.swissadme.ch/, accessed on 8 August 2025) and pkCSM (http://biosig.unimelb.edu.au/pkcsm/, accessed on 8 August 2025) [34]. Canonical SMILES representations of the compounds were obtained from the PubChem database and used as input for the predictions. SwissADME was employed to evaluate key physicochemical properties, including lipophilicity, topological polar surface area (TPSA), aqueous solubility, and drug-likeness based on Lipinski’s Rule of Five [35] and Ghose’s filter [36]. Additionally, pkCSM was used to predict multiple ADMET-related parameters, such as Caco-2 cell permeability, human intestinal absorption, BBB permeability, volume of distribution, cytochrome (CYP) 450 enzyme inhibition profiles, total clearance, and various toxicity endpoints including AMES mutagenicity, hepatotoxicity, acute oral toxicity median lethal dose 50 (LD_50_), and chronic oral toxicity Lowest observed adverse effect level (LOAEL) in rats.

### 2.8. Statistical Analysis

Statistical differences among groups were assessed using one-way analysis of variance (ANOVA), followed by Tukey’s post hoc multiple-comparison test. In all cases, a *p* value < 0.05 was considered statistically significant. Outlier analysis was performed using the ROUT (robust regression and outlier removal) method (Q = 1%), and identified outliers were excluded from analysis. All analyses were conducted using GraphPad Prism 7 (version 7.02, GraphPad Software, San Diego, CA, USA). Animals that died within 24 h after stroke induction were excluded from the corresponding analyses (CMC group, *n* = 3; FA 300 mg/kg, *n* = 1; FAD 300 mg/kg, *n* = 1).

## 3. Results

### 3.1. FAD012 Attenuates Photothrombosis-Induced Reduction in CBF

**Figure 3:**
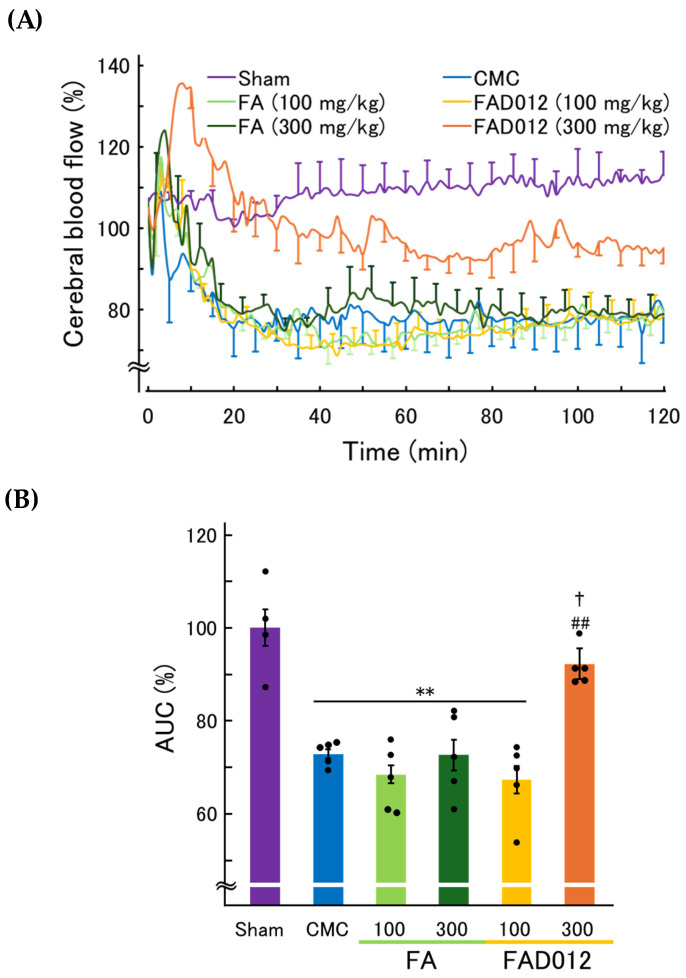
FAD012 prevents the reduction in cerebral blood flow (CBF) following photothrombotic stroke in rats. (**A**) Time course of changes in cortical CBF in the left parietal cortex after laser irradiation. CBF was measured by two-dimensional laser Doppler flowmetry for 120 min following stroke induction. Rats received intraperitoneal injection of FA or FAD012 (100 or 300 mg/kg) 60 min before photothrombosis. (**B**) Area under the curve (AUC) for CBF from 40 to 120 min after stroke induction. Data are presented as mean ± SEM (*n* = 4–5). ** *p* < 0.01 vs. Sham; ## *p* < 0.01 vs. CMC, † *p* < 0.05 vs. FA.

To assess the effects of FAD012 on cerebral perfusion following photothrombotic stroke in rats, CBF in the infarct hemisphere (left parietal cortex) was continuously monitored for 120 min using laser Doppler flowmetry (Figure 3A). In the CMC group, CBF markedly declined beginning approximately 10 min after Rose Bengal administration, indicating successful thrombus formation and the onset of ischemia. Comparable reductions in CBF were observed in the groups treated with FA at 100 or 300 mg/kg and with FAD012 at 100 mg/kg, suggesting that treatment with FA or FAD012 at these doses was insufficient to preserve perfusion. In contrast, rats receiving FAD012 at 300 mg/kg exhibited a sustained attenuation of CBF decline, maintaining relatively stable blood flow throughout the observation period.

Quantitative analysis of the area under the curve (AUC) from 40 to 120 min post-irradiation (Figure 3B) confirmed that FAD012 at 300 mg/kg significantly preserved CBF compared to the CMC group (*p* < 0.01). No significant improvement was observed with FA at 300 mg/kg or with either compound at 100 mg/kg compared to the CMC group. These findings suggest that FAD012 confers dose-dependent protection against ischemia-induced hypoperfusion.

The elapsed time to CBF reduction, defined as the time from photothrombosis onset (Rose Bengal injection) to the nadir of CBF, was significantly prolonged in the FAD012 (300 mg/kg) group compared with the CMC group (*p* < 0.01) and the FA (300 mg/kg) group (*p* < 0.05) (Table 1).

### 3.2. Neuroprotective Effects of FAD012 Assessed by TTC Staining

**Figure 4:**
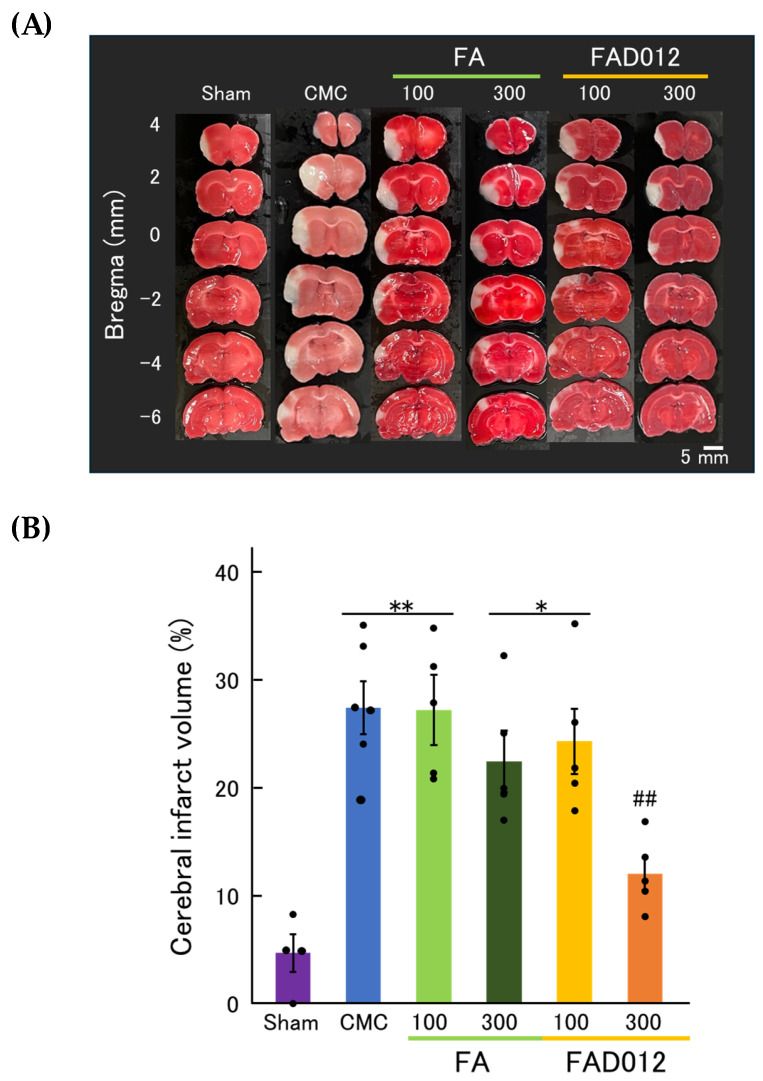
(**A**) Representative 2,3,5-triphenyl tetrazolium chloride (TTC)-stained coronal brain sections at different Bregma levels (from +4 mm to –6 mm) in each treatment group after photothrombotic stroke. Pale areas indicate infarcted regions. (**B**) Quantification of infarct volume in each group. Data are presented as mean ± SEM (*n* = 4–6). *, ** *p* < 0.05, 0.01 vs. Sham; ## *p* < 0.01 vs. CMC.

As shown in the TTC-stained coronal brain sections (Figure 4A), the CMC group exhibited extensive infarction in the cortical regions supplied by the laser-irradiated middle cerebral artery, reflecting severe ischemic injury. Likewise, the FA (100 mg/kg) and FAD012 (100 mg/kg) groups exhibited large infarct areas comparable to those observed in the CMC group, indicating that low-dose administration of either compound did not confer significant neuroprotection. In contrast, the FAD012 (300 mg/kg) group demonstrated a marked reduction in infarct area across the coronal sections, particularly in the cortex and striatum. The FA (300 mg/kg) group exhibited a moderate decrease in infarct size; however, the extent of protection was inferior to that observed in the FAD012 (300 mg/kg) group. Quantitative analysis of infarct volume (Figure 4B) was consistent with these observations. The CMC group exhibited significantly larger infarct volumes than the sham group (** *p* < 0.01). Neither FA (100 mg/kg) nor FAD012 (100 mg/kg) significantly reduced infarct volume relative to the CMC group. Although FA (300 mg/kg) showed a modest reduction in infarct volume, the difference did not reach statistical significance. In contrast, FAD012 (300 mg/kg) significantly attenuated infarct volume compared to CMC (*p* < 0.01), suggesting that FAD012 exerts a more potent neuroprotective effect than its parent compound, FA.

### 3.3. Preservation of BBB Integrity by FAD012 in Ischemic Stroke

**Figure 5:**
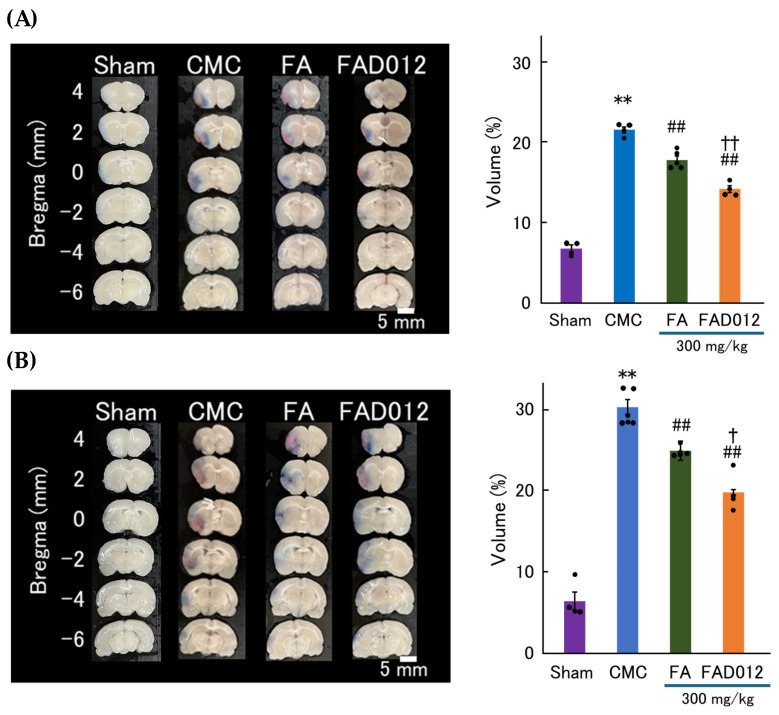
Protective effects of FAD012 on blood–brain barrier (BBB) integrity after photothrombotic stroke. Evans Blue (EB) extravasation was used to assess BBB disruption at 6 and 24 h after stroke induction in rats treated with 300 mg/kg of FA or FAD012. (**A**) Representative coronal brain sections and quantification of EB leakage at 6 h post-stroke. Both FA and FAD012 reduced BBB disruption compared to the CMC group, with FAD012 showing a greater effect. (**B**) Representative coronal brain sections and quantification at 24 h post-stroke. FAD012 maintained its protective effect on BBB integrity. Data are expressed as mean ± SEM (*n* = 4–6). ** *p* < 0.01 vs. Sham; ## *p* < 0.01 vs. CMC; †, †† *p* < 0.05, 0.01 vs. FA.

The extent of BBB disruption was evaluated at 6 and 24 h after photothrombotic stroke induction using EB extravasation (Figure 5). These experiments were conducted using FA and FAD012 at 300 mg/kg, the dose that showed differential neuroprotective effects between the two compounds in the TTC staining analysis. At 6 h post-stroke, the FA-treated group exhibited a significant reduction in BBB disruption volume compared to the CMC group, indicating a protective effect on vascular integrity. Notably, the FAD012-treated group demonstrated a significantly greater reduction in BBB disruption volume relative to the FA group, suggesting superior efficacy (Figure 5A). At 24 h post-stroke, FAD012 treatment continued to markedly suppress BBB disruption, supporting its sustained vascular protective effects (Figure 5B). Collectively, these findings indicate that FAD012 not only attenuates ischemic brain injury but also provides sustained protection of BBB integrity during the acute phase of ischemic stroke.

**Figure 6:**
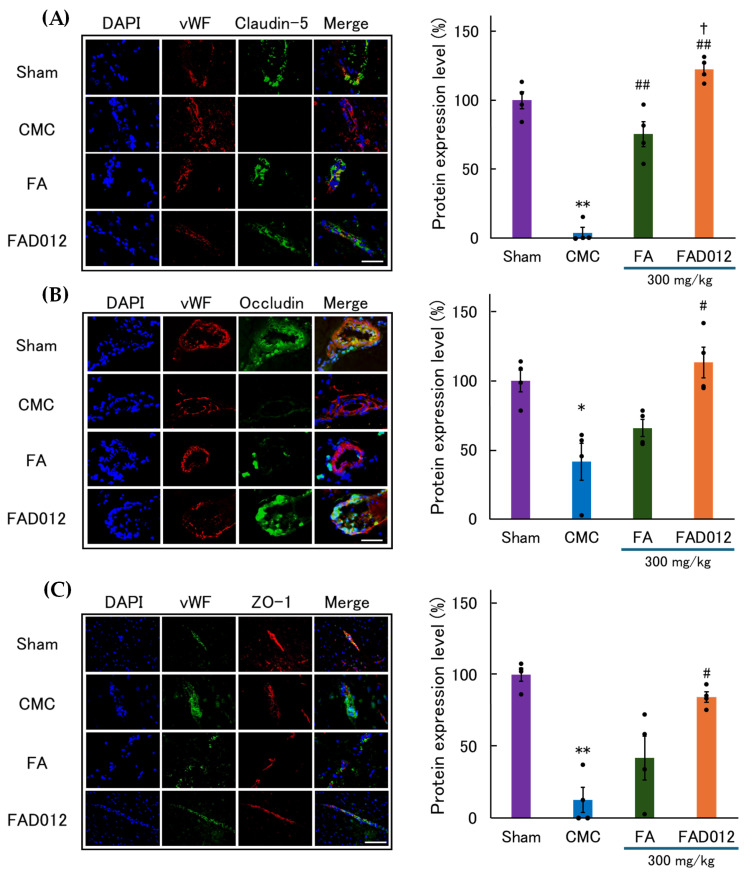
Representative immunofluorescence images and quantification of tight junction (TJ) proteins in the ischemic cortex 24 h after photothrombotic stroke in rats treated with 300 mg/kg of FA or FAD012. (**A**) Claudin-5 (green), (**B**) Occludin (green), (**C**) ZO-1 (red). In each set, von Willebrand factor (vWF) was co-stained as an endothelial marker (fluorescence color varied depending on antibody combinations: red or green), and nuclei were counterstained with 4′,6-Diamidino-2-phenylindole (DAPI: blue). Merged images show co-localization of TJ proteins with vWF. Scale bar = 50 μm. Right panels: Quantification of the positive area for each marker (% of total field, mean ± SEM, *n* = 4 per group). *, ** *p* < 0.05, 0.01 vs. Sham; #, ## *p* < 0.05, 0.01 vs. CMC; † *p* < 0.05 vs. FA.

Immunofluorescence analysis was conducted to assess the effects of 300 mg/kg of FA or FAD012 on TJ proteins in the penumbral cortex 24 h after photothrombotic stroke (Figure 6). In the CMC group, the immunoreactivity of claudin-5, occludin, and ZO-1 was markedly reduced along the vascular endothelium, indicating BBB disruption. Treatment with FA partially restored the expression of these proteins. In contrast, FAD012 preserved continuous endothelial localization of TJ proteins, showing robust linear staining patterns. Quantitative analysis confirmed that the immunoreactivity of claudin-5, occludin, and ZO-1 was significantly higher in the FAD012 group compared with both the CMC and FA groups (Figure 6, right panel).

**Figure 7:**
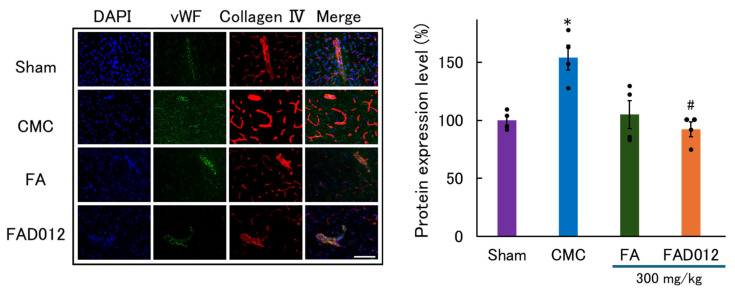
Representative immunofluorescence images and quantification of collagen IV expression in the ischemic cortex 24 h after photothrombotic stroke in rats treated with 300 mg/kg of FA or FAD012. Collagen IV (red) was co-stained with vWF (green) as an endothelial marker, and nuclei were counterstained with DAPI (blue). Quantitative analysis of fluorescence intensity is shown in the right panel. Data are presented as mean ± SEM (*n* = 4 per group). Scale bar = 50 μm. * *p* < 0.05 vs. Sham; # *p* < 0.05 vs. CMC.

Collagen IV, a major component of the vascular basement membrane, exhibited abnormally enhanced and fragmented staining in the CMC group, indicating ischemia-induced disruption and remodeling of the basement membrane. FA (300 mg/kg) treatment partially alleviated these changes. In contrast, FAD012 (300 mg/kg) markedly suppressed the abnormal increase in collagen IV intensity and restored its continuous perivascular localization, suggesting preserved basement membrane integrity (Figure 7). Quantitative analysis further demonstrated that FAD012 significantly attenuated the pathological upregulation of collagen IV compared with the CMC group (Figure 7, right panel).

### 3.4. Involvement of NO in the CBF-Preserving Effect of FAD012

**Figure 8:**
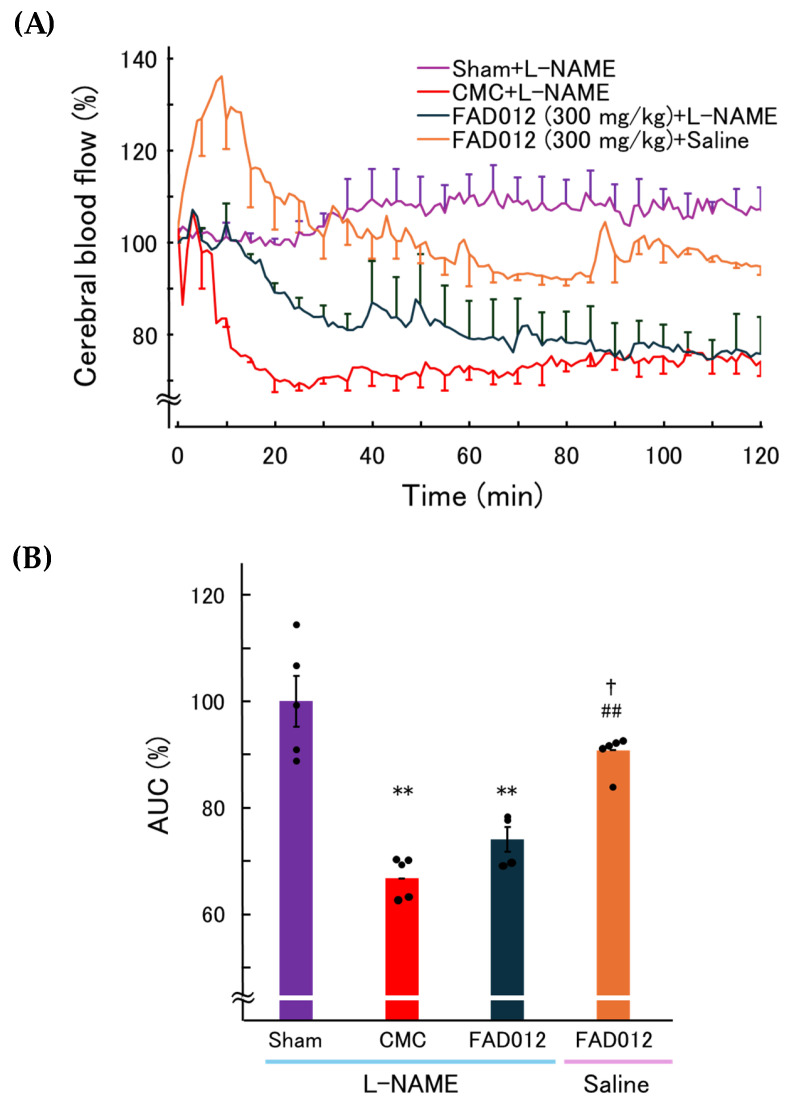
Effect of L-NAME pretreatment on CBF dynamics after photothrombotic stroke. (**A**) Time course of relative CBF (% of baseline) measured by laser Doppler flowmetry in four groups: Sham + L-NAME (3 mg/kg), CMC + L-NAME, FAD012 (300 mg/kg) + L-NAME, and FAD012 (300 mg/kg) + saline (*n* = 4–5 per group). Photothrombotic stroke was induced by intravenous injection of Rose Bengal at 10 min, followed by green laser irradiation. (**B**) AUC for CBF from 40 to 120 min after stroke induction. Data are presented as mean ± SEM (*n* = 4–5). ** *p* < 0.01 vs. Sham; ## *p* < 0.01 vs. CMC, † *p* < 0.05 vs. FAD012 + L-NAME.

The role of NO in the action of FAD012 (300 mg/kg) to preserve CBF during ischemia was assessed using L-NAME, a selective NOS inhibitor, commonly employed to investigate the involvement of NO signaling (Figure 8). L-NAME (3 mg/kg), which alone had no effect on CBF, was administered intraperitoneally 30 min prior to the induction of photothrombotic stroke. Pretreatment with L-NAME completely abolished the CBF-preserving effect of FAD012, indicating that the maintenance of CBF by FAD012 under ischemic conditions depends on NO signaling pathways. In contrast, the elapsed time to CBF nadir in the FAD012 group was not significantly altered by pretreatment with L-NAME (79.0 ± 6.2 min with L-NAME vs. 79.0 ± 3.9 min with saline).

**Figure 9:**
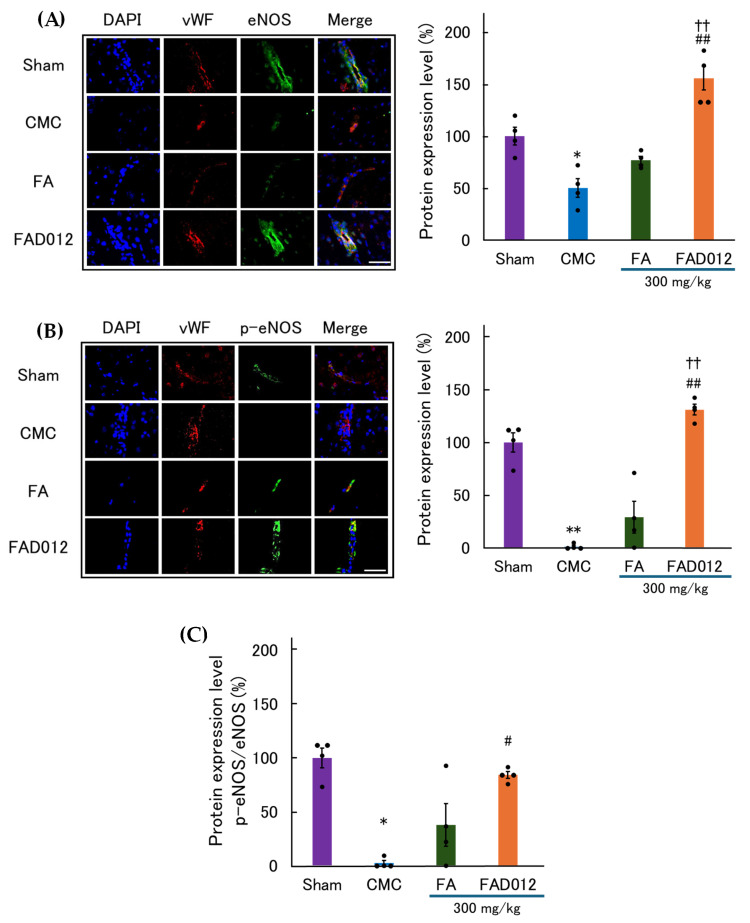
Representative immunofluorescence images and quantification of endothelial total endothelial nitric oxide synthase (eNOS) and phosphorylated eNOS (p-eNOS) in the ischemic cortex 24 h after photothrombotic stroke. (**A**) Total eNOS (green) co-localized with endothelial cells identified by vWF (red) in the peri-infarct cortex of rats treated with CMC, FA (300 mg/kg), or FAD012 (300 mg/kg). The graph shows the total eNOS–positive area as a percentage of the total field (mean ± SEM, *n* = 4 per group). (**B**) p-eNOS (green) co-localized with vWF (red) in the same groups as in (A). The graph shows the p-eNOS–positive area (% of total field, mean ± SEM, *n* = 4 per group). All sections were counterstained with DAPI (blue) to visualize nuclei. Merged images illustrate endothelial localization of eNOS and p-eNOS. Scale bar = 50 μm. (**C**) Ratio of p-eNOS to total eNOS, calculated from the data in (A) and (B), as an index of endothelial eNOS activation (mean ± SEM, *n* = 4 per group). *, ** *p* < 0.05, 0.01 vs. Sham; #, ## *p* < 0.05, 0.01 vs. CMC; †† *p* < 0.01 vs. FA.

Immunofluorescence staining was performed to assess the expression of total eNOS and its p-eNOS (phosphorylated form at Ser1177), an activation-associated site, in the penumbral cortex 24 h after photothrombotic stroke (Figure 9). In the CMC group, eNOS immunoreactivity was attenuated and distributed sporadically along the vascular endothelium, and p-eNOS staining was similarly reduced. In contrast, rats pretreated with FAD012 (300 mg/kg) exhibited a marked enhancement in both eNOS and p-eNOS immunoreactivities, with clear co-localization with vWF, indicating upregulation and activation of endothelial eNOS. Quantitative analysis confirmed that eNOS and p-eNOS expression, as well as the p-eNOS/eNOS ratio reflecting the activation state of eNOS, were all significantly higher in the FAD012 group compared with both the CMC and FA groups. These results suggest that FAD012 not only preserves eNOS expression abundance but also promotes its phosphorylation at Ser1177, thereby supporting sustained NO signaling in the ischemic brain.

### 3.5. Absorption, Distribution, Metabolism, Excretion, and Toxicity Properties of FAD012

To further characterize the pharmacological profile of FAD012, in silico predictions of ADMET were compared with those of its parent compound FA (Table 2). FAD012 exhibited higher lipophilicity (consensus LogP: 2.03 vs. 1.36) and a lower TPSA (57.53 Å^2^ vs. 66.76 Å^2^) than FA, both of which are favorable for passive membrane permeability. Consistently, Caco-2 cell permeability and skin permeation values were higher for FAD012, whereas predicted intestinal absorption was similarly high for both compounds (>95%). Both FA and FAD012 satisfied established drug-likeness criteria, including Lipinski’s “Rule of Five” [35] and Ghose’s filter [36], indicating favorable physicochemical properties for oral bioavailability. Predicted BBB permeability (log BB) and CNS penetration (log PS) were slightly greater for FAD012 than FA. Predicted toxicity profiles were comparable between the two compounds, with neither showing AMES toxicity, hepatotoxicity, or skin sensitization. Notably, the maximum dose used in this study (300 mg/kg) was far below the predicted acute toxicity threshold. Collectively, these properties suggest that FAD012 possesses favorable pharmacokinetic characteristics without increasing predicted toxicity relative to FA.

## 4. Discussion

In this study, we demonstrated that FAD012, a synthetic derivative of FA, confers neurovascular protection in a rat photothrombotic stroke model. Pretreatment with FAD012 at 300 mg/kg significantly attenuated the reduction in CBF (Figure 3), reduced infarct volume (Figure 4), and mitigated BBB disruption (Figure 5). These effects were accompanied by preservation of TJ proteins (claudin-5, occludin, and ZO-1) (Figure 6), basement membrane integrity (collagen IV) (Figure 7), and sustained expression and phosphorylation of eNOS (Figure 9). The CBF-preserving effect of FAD012 was completely abolished by pretreatment with the NOS inhibitor L-NAME, indicating that this action is mediated through NO-dependent mechanisms (Figure 8). These protective effects were more pronounced than those observed with the parent compound FA, highlighting the superior efficacy of FAD012. Furthermore, FAD012, like FA, exhibits low toxicity (Table 2), supporting its suitability for long-term prophylactic administration as a preventive strategy against cerebrovascular injury.

The superior efficacy of FAD012 compared to FA may be attributed to its optimized physicochemical properties (Table 2), which enhance its biological availability and membrane-targeting potential. Structurally, FAD012 features 3,5-dimethyl substitutions on the phenyl ring (Figure 1). These substitutions increase lipophilicity and reduce polarity, as indicated by a higher consensus Log *p* value and a lower topological polar surface area, parameters known to influence passive diffusion across biological membranes [35]. Predicted values for skin permeation and Caco-2 cell permeability also favor FAD012, supporting the notion that it more efficiently traverses lipid bilayers compared to FA. Our previous study demonstrated that FAD012 possesses radical scavenging and lipid peroxidation inhibitory activities comparable to or slightly stronger than those of FA and Trolox, a water-soluble vitamin E analog [15]. The incorporation of methyl groups at the 3 and 5 positions of the aromatic ring is expected to improve the electron-donating capacity of the phenolic hydroxyl group, thereby enhancing its antioxidant reactivity. Given that endothelial cells are highly susceptible to oxidative stress [37], such improved reactivity, together with efficient membrane localization, may underlie the superior ability of FAD012 to preserve TJ integrity, prevent BBB breakdown, and maintain cerebral perfusion after ischemia [38]. Furthermore, consistent with the in silico ADMET predictions indicating low toxicity, our previous in vivo study showed that chronic administration of FAD012 did not induce any abnormalities in body weight, hematological indices, or serum biochemical parameters, supporting its favorable safety profile and suitability for long-term prophylactic use [15,35].

A key finding of this study is the preservation of BBB integrity by FAD012 under ischemic conditions (Figure 5, Figure 6 and Figure 7). Disruption of the BBB is a well-recognized pathological feature of acute ischemic stroke and contributes substantially to the progression of neuronal injury. Ischemia-induced oxidative stress, inflammation, and activation of matrix metalloproteinases (MMPs) degrade TJ proteins and basement membrane components, thereby increasing barrier permeability and promoting neuroinflammation [39,40]. FAD012 preserved the expression and localization of claudin-5, occludin, and ZO-1 (Figure 6), indicating that it maintains the structural integrity of the endothelial barrier. Preservation of BBB integrity is essential for restricting paracellular leakage, maintaining ionic homeostasis, limiting immune cell infiltration, and preserving the extracellular microenvironment required for neuronal survival [39]. These protective effects of FAD012 on BBB components likely account, at least in part, for the reduction in infarct size. In addition, FAD012 suppressed the fragmentation of collagen IV, a major basement membrane protein (Figure 7). Collagen IV degradation is a hallmark of BBB disruption under ischemic conditions [41] and is frequently mediated by MMPs, which are activated in response to oxidative stress [42,43]. Preservation of collagen IV integrity by FAD012 suggests stabilization of the extracellular matrix and basal lamina, which may prevent endothelial detachment, vasogenic edema, and hemorrhagic transformation during acute ischemia.

Oxidative stress, in particular, represents a critical driver of BBB injury. Excessive ROS production occurs rapidly after vascular occlusion and is closely linked to stroke-induced inflammation. In this context, the antioxidant activity of FAD012 is likely to play a decisive role in its BBB-protective effect. By scavenging ROS and inhibiting lipid peroxidation, FAD012 may attenuate the cascade of oxidative endothelial damage that leads to TJ protein disassembly, and basement membrane degradation. Importantly, FAD012 delayed the decline in CBF in the photothrombotic model (Figure 3 and Figure 8), suggesting suppression of endothelial injury and thrombus formation. Because photochemically induced thrombosis is primarily driven by ROS-mediated endothelial injury and platelet activation [29], it is plausible that the antioxidative properties of FAD012—through both direct radical scavenging and suppression of oxidative signaling—play a central role in attenuating thrombus formation. FA has also been reported to activate the nuclear factor erythroid 2–related factor 2 (Nrf2)/heme oxygenase-1 (HO-1) antioxidant pathway, enhancing cellular defense by promoting Nrf2 nuclear translocation and upregulating HO-1 expression in neuronal and epithelial cells [44]. Although ROS levels were not directly measured in this study, the antioxidative properties of FAD012 have been demonstrated previously in vivo using a chronic cerebral hypoperfusion model (2VO) [16]. Supporting this notion, our recent study using RBMVECs demonstrated that FAD012 markedly upregulates Nrf2 expression and induces HO-1 (Aoyama et al., in preparation). These responses were accompanied by reduced intracellular ROS and improved cell viability under oxidative stress, whereas pharmacological inhibition of Nrf2 abolished the protective effects. Given the central role of Nrf2 in redox homeostasis and endothelial integrity [45], its activation by FAD012 may contribute to the preservation of TJ proteins, suppression of BBB disruption, and mitigation of ROS-driven platelet activation and thrombus formation.

Another key finding of this study is that the CBF-preserving effect of FAD012 under ischemic conditions was abolished by pretreatment with L-NAME, a NOS inhibitor (Figure 8). This observation strongly suggests that the maintenance of CBF by FAD012 is mediated by NO-dependent mechanisms. Supporting this, immunofluorescence analysis revealed that FAD012 preserved both total eNOS expression and its p-eNOS (phosphorylated form) in the peri-infarct vasculature. These results are in agreement with our previous findings in a MCAO/Re model, where FAD012 maintained eNOS expression and attenuated ischemic injury [15], as well as in a chronic cerebral hypoperfusion model [16]. The preservation of eNOS expression and its phosphorylation after FAD012 treatment may reflect the protection of endothelial cells from ischemic injury, rather than a direct transcriptional upregulation. Given that eNOS phosphorylation can occur within minutes of stimulation [46], it is plausible that FAD012 enhances NO production early after ischemia onset. eNOS-derived NO plays a pivotal role in maintaining cerebral perfusion by promoting vasodilation and inhibiting platelet aggregation, both of which are critical during the early phase of stroke when cerebral autoregulatory mechanisms are compromised [47]. Under oxidative stress, however, NO reacts with superoxide (O_2_^−^·) to form peroxynitrite, a potent oxidant that not only impairs eNOS function and reduces NO bioavailability, but also induces endothelial injury and promotes MMP-9 activation, leading to BBB disruption [48]. While some experimental studies have suggested beneficial effects of NO donors in stroke models [49,50], their clinical utility remains uncertain. Thus, the relative dominance of NO signaling versus oxidative stress critically determines vascular outcome after ischemia. By sustaining eNOS expression and attenuating oxidative stress, FAD012 may contribute to preserve NO bioavailability, limit peroxynitrite formation, and maintain vascular tone and microcirculatory flow. A limitation of this study is the absence of direct, real-time measurements of NO production and microvascular tone in cerebral microvessels. Future work will complement the present findings with targeted assays to directly quantify NO signaling and vascular reactivity.

Although the precise upstream mechanisms responsible for eNOS phosphorylation by FAD012 remain unclear, FA and its derivatives have been shown to activate key endothelial signaling pathways such as phosphoinositide 3-kinase (PI3K)/Akt and AMP-activated protein kinase (AMPK), both of which enhance eNOS activity via Ser1177 phosphorylation [51,52,53]. FA has also been reported to increase eNOS expression and NO production in experimental models of cerebral ischemia, likely through antioxidative mechanisms [54]. Given that FAD012 is a structurally modified derivative of FA with increased lipophilicity and membrane permeability, it is plausible that it may more efficiently engage these intracellular signaling pathways, resulting in enhanced endothelial resilience and eNOS activation. Further studies are warranted to determine whether FAD012 directly activates PI3K/Akt, AMPK, or other kinases involved in endothelial function [54].

Previous studies have variably reported the brain penetration of FA. Some demonstrated rapid detection in brain tissue within minutes after systemic administration [55], whereas others indicated limited exposure following intraperitoneal injection [56]. Nonetheless, the primary therapeutic target in the present study is the cerebral microvascular endothelium, and therefore extensive parenchymal penetration is not a prerequisite for the observed protective effects. We recognize that comprehensive pharmacokinetic studies remain necessary, and these investigations are currently in progress to clarify plasma and brain concentrations as well as the metabolic fate of FAD012.

In this study, our main purpose was to evaluate FAD012 as a preventive therapeutic agent. Our ongoing studies have confirmed protective effects of repeated low-dose FAD012 administration (30 mg/kg administered for 1 week, Sugoh et al., manuscript in preparation), further supporting its prophylactic potential. Future studies will also investigate the combination of FAD012 with established therapeutic agents such as edaravone and tPA to assess its translational applicability.

The present study provides several novel insights beyond previous investigations using MCAO or 2VO models [15,16]. By employing the photothrombotic stroke model, which more closely reflects clinical thrombotic events, we demonstrated for the first time that FAD012 suppresses thrombus formation associated with endothelial stress injury and preserves BBB integrity. In particular, FAD012 maintained the expression of tight junction proteins and basement membrane integrity. Moreover, this study newly shows that FAD012 enhances eNOS phosphorylation, indicating activation of eNOS signaling in addition to preserving total eNOS expression. Collectively, these findings highlight the unique prophylactic potential of FAD012 in clinically relevant thrombotic conditions.

## 5. Conclusions

Intraperitoneal pretreatment with FAD012 attenuated ischemic brain injury in the photothrombotic stroke rat model by preserving cerebral perfusion, maintaining endothelial function, and protecting BBB integrity. The CBF-preserving effect was demonstrated to be mediated by NO-dependent mechanisms, as evidenced by its abolition with NOS inhibition. In addition, the antioxidative properties of FAD012 are likely to contribute to the suppression of thrombus formation and the preservation of TJ proteins and basement membrane structure. Together with its favorable safety profile, these findings suggest that FAD012 is a promising low-toxicity candidate for prophylactic administration in individuals at high risk of stroke, as well as a potential adjunctive agent in therapeutic strategies for ischemic cerebrovascular disease.

## Figures and Tables

**Table 1:** Elapsed time to CBF nadir following photothrombotic stroke in rats treated with FA or FAD012.

Groups	Elapsed Times to CBF Nadir (Min)
CMC	30.6 ± 3.8
FA (100 mg/kg)	42.8 ± 7.5
FA (300 mg/kg)	36.6 ± 2.8
FAD012 (100 mg/kg)	50.2 ± 7.1
FAD012 (300 mg/kg)	70.6 ± 1.6 **^,^†

Values represent the elapsed time from stroke induction to the nadir of CBF, measured by laser Doppler flowmetry. Data are expressed as mean ± SEM (*n* = 5). ** *p* < 0.01 vs. CMC; † *p* < 0.05 vs. FA (300 mg/kg) (one-way analysis of variance (ANOVA) with post hoc test).

**Table 2:** Pharmacokinetic properties and absorption, distribution, metabolism, excretion, and toxicity (ADMET) prediction of FAD012 using SwissADME and pkCSM.

	FAD012	FA
Physicochemical Properties		
Formula	C_10_H_10_O_4_	C_11_H_12_O_3_
Molecular weight	192.214	194.186
Topological Polar Surface Area (TPSA) (Å^2^)	57.53	66.76
Consensus LogP	2.03	1.36
Water solubility (log mol/L)	−2.57	−2.81
Number of H-bond acceptors	3	4
Number of H-bond donors	2	2
Lipinski’s rule	Yes; 0 violation	Yes; 0 violation
Ghose’s rule	Yes	Yes
Absorption		
Caco-2 permeability (log Papp in 10^−6^ cm/s)	1.20	0.19
Intestinal absorption (human)(% Absorbed)	97.2	95.7
Skin permeation (log Kp; cm/s)	−5.83	−6.41
P-glycoprotein substrate	Yes	Yes
P-glycoprotein I inhibitor	No	No
P-glycoprotein II inhibitor	No	No
Distribution		
BBB permeability (log BB)	−0.17	−0.30
CNS permeability (log PS)	−2.83	−2.55
Volume of distribution (log L/kg)	−0.79	−0.90
Metabolism		
CYP2D6 substrate	No	No
CYP3A4 substrate	No	No
CYP1A2 inhibitor	No	No
CYP2C9 inhibitor	No	No
CYP2C9 inhibitor	No	No
CYP2D6 inhibitor	No	No
CYP3A4 inhibitor	No	No
Excretion		
Total Clearance (log ml/min/kg)	0.72	0.64
Renal OCT2 substrate	No	No
Toxicity		
AMES toxicity	No	No
Max. tolerated dose (human)(log mg/kg/day) (mg/kg/day, calculated from log mol/kg)	1.70 (50.1)	1.49 (30.9)
Oral Rat Acute Toxicity (LD_50_)(mol/kg) (mg/kg, calculated from log mol/kg)	2.39 (4.7 × 10^7^)	2.52 (6.4 × 10^7^)
Oral Rat Chronic Toxicity (LOAEL)(log mg/kg_bw/day) (mg/kg_bw/day, calculated from log mol/kg_bw/day)	2.30 (200)	1.94 (87.1)
Hepatotoxicity	No	No
Skin Sensitization	No	No

## Data Availability

The data presented in this study are available on request from the corresponding author.

## Abbreviations

The following abbreviations are used in this manuscript:

ADMET	Absorption, distribution, metabolism, excretion, and toxicity
AMPK	AMP-activated protein kinase
ANOVA	Analysis of variance
AUC	Area under the curve
BBB	Blood–brain barrier
CBF	Cerebral blood flow
CMC	Carboxymethyl cellulose
CNS	Central nervous system
CYP	Cytochrome pigment
DAPI	4′,6-Diamidino-2-phenylindole
EB	Evans blue
eNOS	endothelial NOS
FA	Ferulic acid; 4-hydroxy-3-methoxycinnamic acid
FAD012	3,5-Dimethyl-4-hydroxy cinnamic acid
HO-1	Heme oxygenase-1
i.p.	Intraperitoneal
i.v.	Intravenous
LD_50_	Lethal dose 50
LOAEL	Lowest observed adverse effect level
L-NAME	N^G^-nitro-L-arginine methyl ester
MCAO/Re	Middle cerebral artery occlusion following reperfusion
MMPs	Matrix metalloproteinases
NO	Nitric oxide
NOS	NO synthase
Nrf2	Nuclear factor erythroid 2-related factor 2
PI3K	phosphoinositide 3-kinase
t-PA	Tissue plasminogen activator
TTC	2,3,5-Triphenyl tetrazolium chloride
PBS	Phosphate-buffered saline
p-eNOS	phosphorylated eNOS
RBMVECs	Rat brain microvascular endothelial cells
ROS	Reactive oxygen species
TJ	Tight junction
vWF	von Willebrand factor
